# Supplementary material for: Spatial information in large-scale neural recordings
Source: Front Comput Neurosci. 2015 Jan 21;8:172. doi: 10.3389/fncom.2014.00172 (PMC4301009; doi:10.3389/fncom.2014.00172)
Supplement: Supplementary file 1 [file DataSheet1.PDF]

1 Supplementary Information

1.1 Noise Sources

The Fisher information framework allows for arbitrary noise sources, so long as they are able to be modeled. However, to demonstrate potential applications, we used a very simplified noise model that only considered signal dependent noise where the standard deviation was proportional to the mean.

There are multiple potentially relevant sources of noise that could readily be included in our model. (1) Each sensor has a constant level of noise simply due to thermal effects. (2) Many sensors have an additional variance that is proportional to the square of the signals, e.g. reference fluctuations. (3) Many sensors have an additional variance that is proportional to the signal, e.g. due to low numbers of photons (shot noise). (4,5) Each neuron may produce constant noise, e.g. background fluorescence of dyes. These neural noise sources may be independent or correlated. (6,7) Each neuron may produce variance that quadratically depends on its activation, e.g. action potentials that propagate back into varying parts of the dendritic tree.[1] These neural noise sources may be independent or correlated. (8,9) Each neuron may produce variance that linearly depends on its signal strength, e.g. fluorophore activations. These neural noise sources may be independent or correlated. We have some knowledge about the exact sizes of these signals [1], but most of these numbers are hard to know. They may be reasonable to measure in future experiments.

Taking these signals together, we obtain the following noise level on a sensor i (given a recording of N firing neurons indexed by j):

[1] In a simplistic model, when a neuron fires, the action potential spreads into some variable proportion of the dendritic tree. If the recorded signal is dependent on the proportion of dendritic branches the action potential propagates into, then the standard deviation of the recorded signal is proportionate to the mean signal entering the dendrites.

$$
\begin{aligned}
{\sigma^2}_{noise} = & \underbrace{\sigma^2_{sens}}_{constant\, sensor\, noise} + \underbrace{\alpha_1 \sum_{j=1}^{N} {I_0}^2 \big(w(\boldsymbol{d}^{i,j})\big)^2}_{ind\, sensor\, \sigma \propto \mu\, SDN} + \underbrace{\alpha_2 \sum_{j=1}^{N} I_0 \big(w(\boldsymbol{d}^{i,j})\big)^2}_{ind\, sensor\, \sigma^2 \propto \mu\, SDN} \\
& + \underbrace{\alpha_3 N \cdot \sigma^2_{neur}}_{constant\, ind\, neuron\, noise} + \underbrace{\alpha_4 N \cdot \sigma^2_{neur}}_{constant\, corr\, neuron\, noise} \\
& + \underbrace{\alpha_5 \sum_{j=1}^{N} {I_0}^2 \big(w(\boldsymbol{d}^{i,j})\big)^2}_{ind\, neuron\, \sigma \propto \mu\, SDN} + \underbrace{\alpha_6 \left(\sum_{j=1}^{N} I_0 \big(w(\boldsymbol{d}^{i,j})\big) \right)^2}_{corr\, neuron\, \sigma \propto \mu\, SDN} \\
& + \underbrace{\alpha_7 \sum_{j=1}^{N} I_0 \big(w(\boldsymbol{d}^{i,j})\big)}_{ind\, neuron\, \sigma^2 \propto \mu\, SDN} + \underbrace{\alpha_8 \left(\sum_{j=1}^{N} \sqrt{I_0 \big(w(\boldsymbol{d}^{i,j})\big)} \right)^2}_{corr\, neuron\, \sigma^2 \propto \mu\, SDN}
\end{aligned} \tag{1}
$$

where *ind* and *corr* refer to independent and correlated noise sources, and SDN refers to signal dependent noise.

Assuming, as we do in the main text's demonstrations, that neurons are uniformly distributed and have a uniform firing rate across the entire volume:

$$
\begin{aligned}
{\sigma^2}_{noise} = & \underbrace{\sigma^2_{sens}}_{constant\, sensor\, noise} + \underbrace{\alpha_1 \rho_{fire} \rho_{space} \int {I_0}^2 w^2 dV}_{ind\, sensor\, \sigma \propto \mu\, SDN} + \underbrace{\alpha_2 \rho_{fire} \rho_{space} \int I_0 w dV}_{ind\, sensor\, \sigma^2 \propto \mu\, SDN} \\
& + \underbrace{\alpha_3 \rho_{fire} \rho_{space} V \cdot \sigma^2_{neur}}_{constant\, ind\, neuron\, noise} + \underbrace{\alpha_4 \rho_{fire} \rho_{space} V \cdot \sigma^2_{neur}}_{constant\, corr\, neuron\, noise} \\
& + \underbrace{\alpha_5 \rho_{fire} \rho_{space} \int {I_0}^2 w^2 dV}_{ind\, neuron\, \sigma \propto \mu\, SDN} + \underbrace{\alpha_6 \rho_{fire} \rho_{space} \left(\int I_0 w dV \right)^2}_{corr\, neuron\, \sigma \propto \mu\, SDN} \\
& + \underbrace{\alpha_7 \rho_{fire} \rho_{space} \int I_0 w dV}_{ind\, neuron\, \sigma^2 \propto \mu\, SDN} + \underbrace{\alpha_8 \rho_{fire} \rho_{space} \left(\int \sqrt{I_0 w} dV \right)^2}_{corr\, neuron\, \sigma^2 \propto \mu\, SDN}
\end{aligned} \tag{2}
$$

Both constant and shot noise terms can be minimized in their effect by optimizing the experimental design, e.g. through good dyes and strong illumination (but see [1]).

In addition, in the main text we assume that the noise is Gaussian, which has also been assumed in previous statistical formulations [2, 3]. This assumption has been shown to be valid for thermal noise and shot noise in some conditions [4, 5].

1.2 Fisher Information Shortcut Derivation

We have assumed that the distribution of signals on a sensor is a Gaussian distribution: $f(X;\boldsymbol{\theta}) = \mathcal{N}\left(\mu\left(\boldsymbol{\theta}\right), \sigma_{noise}^2\right)$, where μ is a function that describes the mean signal as a function of the parameters in $\boldsymbol{\theta}$. In practice, $\boldsymbol{\theta}$ represents the positions and intensities of the various neurons being analyzed, and $\mu\left(\cdot\right)$ is some representation of the PSFs that relate signals to sensors. Due to this Gaussian assumption, solving for the Fisher information matrix simplifies:

$$\mathcal{I}_{jk} = E\left[\left(\frac{\partial}{\partial\theta_j} ln\left(f\left(X;\boldsymbol{\theta}\right)\right)\right)\left(\frac{\partial}{\partial\theta_k} ln\left(f\left(X;\boldsymbol{\theta}\right)\right)\right)\right] \tag{3}$$

$$\begin{aligned}\frac{\partial}{\partial\theta_j} ln\left(f\left(X;\boldsymbol{\theta}\right)\right) =& \frac{1}{f\left(X;\boldsymbol{\theta}\right)}\frac{\partial}{\partial\theta_j} f\left(X;\boldsymbol{\theta}\right)\\ =& \frac{1}{\frac{1}{\sqrt{2\pi\sigma_{noise}^2}}\exp\left(\frac{-(X-\mu(\boldsymbol{\theta}))^2}{2\sigma_{noise}^2}\right)}\cdot\\ & \frac{1}{\sqrt{2\pi\sigma_{noise}^2}}\exp\left(\frac{-\left(X-\mu\left(\boldsymbol{\theta}\right)\right)^2}{2\sigma_{noise}^2}\right)\cdot\\ & \frac{\partial}{\partial\theta_j}\left(\frac{-\left(X-\mu\left(\boldsymbol{\theta}\right)\right)^2}{2\sigma_{noise}^2}\right)\\ =& \frac{1}{2\sigma_{noise}^2}\cdot 2\left(X-\mu\left(\boldsymbol{\theta}\right)\right)\cdot\frac{\partial\mu\left(\boldsymbol{\theta}\right)}{\partial\theta_j}\\ =& \frac{\left(X-\mu\left(\boldsymbol{\theta}\right)\right)}{\sigma_{noise}^2}\frac{\partial\mu\left(\boldsymbol{\theta}\right)}{\partial\theta_j}\end{aligned} \tag{4}$$

$$
\begin{aligned}
\mathcal{I}_{jk} =& E\left[\frac{\left(X-\mu\left(\boldsymbol{\theta}\right)\right)^2}{\sigma_{noise}^4}\frac{\partial\mu\left(\boldsymbol{\theta}\right)}{\partial\theta_j}\frac{\partial\mu\left(\boldsymbol{\theta}\right)}{\partial\theta_k}\right] \\
=& E\left[\frac{\left(X-\mu\left(\boldsymbol{\theta}\right)\right)^2}{\sigma_{noise}^4}\right]\frac{\partial\mu\left(\boldsymbol{\theta}\right)}{\partial\theta_j}\frac{\partial\mu\left(\boldsymbol{\theta}\right)}{\partial\theta_k} \\
=& \frac{1}{\sigma_{noise}^2}\frac{\partial\mu\left(\boldsymbol{\theta}\right)}{\partial\theta_j}\frac{\partial\mu\left(\boldsymbol{\theta}\right)}{\partial\theta_k}
\end{aligned} \tag{5}
$$

2 References

[1] Adam H Marblestone, Bradley M Zamft, Yael G Maguire, Mikhail G Shapiro, Thaddeus R Cybulski, Joshua I Glaser, Dario Amodei, Ben Stranges, Reza Kalhor, David A Dalrymple, Dongjin Seo, Elad Alon, Michel M Maharbiz, Jose M Carmena, Jan M Rabaey, Edward S Boyden, George M Church, and Konrad P Kording. Physical principles for scalable neural recording. *Front Comput Neurosci*, 7:137, 2013.

[2] Morteza Shahram. *Statistical and information-theoretic analysis of resolution in imaging and array processing*. Thesis, 2005.

[3] Morteza Shahram and Peyman Milanfar. Statistical and information-theoretic analysis of resolution in imaging. *Information Theory, IEEE Transactions on*, 52(8):3411–3437, 2006.

[4] Robert Tyson. *Adaptive optics engineering handbook*, volume 67. CRC Press, 1999.

[5] Alan C Bovik. *Handbook of image and video processing*. Access Online via Elsevier, 2010.
